# Supplementary material for: Discovery of Three Toxic Proteins of Klebsiella Phage fHe-Kpn01
Source: Viruses. 2020 May 15;12(5):544. doi: 10.3390/v12050544 (PMC7291057; doi:10.3390/v12050544)
Supplement: Supplementary file 1 [file viruses-12-00544-s001.pdf]

viruses

MDPI

Supplementary information

Discovery of three toxic proteins of Klebsiella phage fHe-Kpn01

Cindy M. Spruit[1,2,†], Anu Wicklund[1,3], Xing Wan[1,4], Mikael Skurnik[1,3], and Maria I. Pajunen[1,*]

1 Department of Bacteriology and Immunology, Medicum, Human Microbiome Research Program, Faculty of Medicine, University of Helsinki, 00290 Helsinki, Finland; c.m.spruit@uu.nl (C.M.S.); anumaria.wicklund@gmail.com (A.W.); xing.wan@helsinki.fi (X.W.); mikael.skurnik@helsinki.fi (M.S.); maria.pajunen@helsinki.fi (M.I.P.)

2 Laboratory of Microbiology, Wageningen University and Research, Wageningen, the Netherlands

3 Division of Clinical Microbiology, HUSLAB, University of Helsinki and Helsinki University Hospital, 00290 Helsinki, Finland

4 Department of Microbiology, Faculty of Agriculture and Forestry, University of Helsinki, 00790 Helsinki, Finland

† Present address: Department of Chemical Biology & Drug Discovery, Utrecht Institute for Pharmaceutical Sciences, Utrecht University, 3584 CG Utrecht, the Netherlands

* Correspondence: maria.pajunen@helsinki.fi; Tel.: +358-50-5677486; OrcID 0000-0001-5484-2228

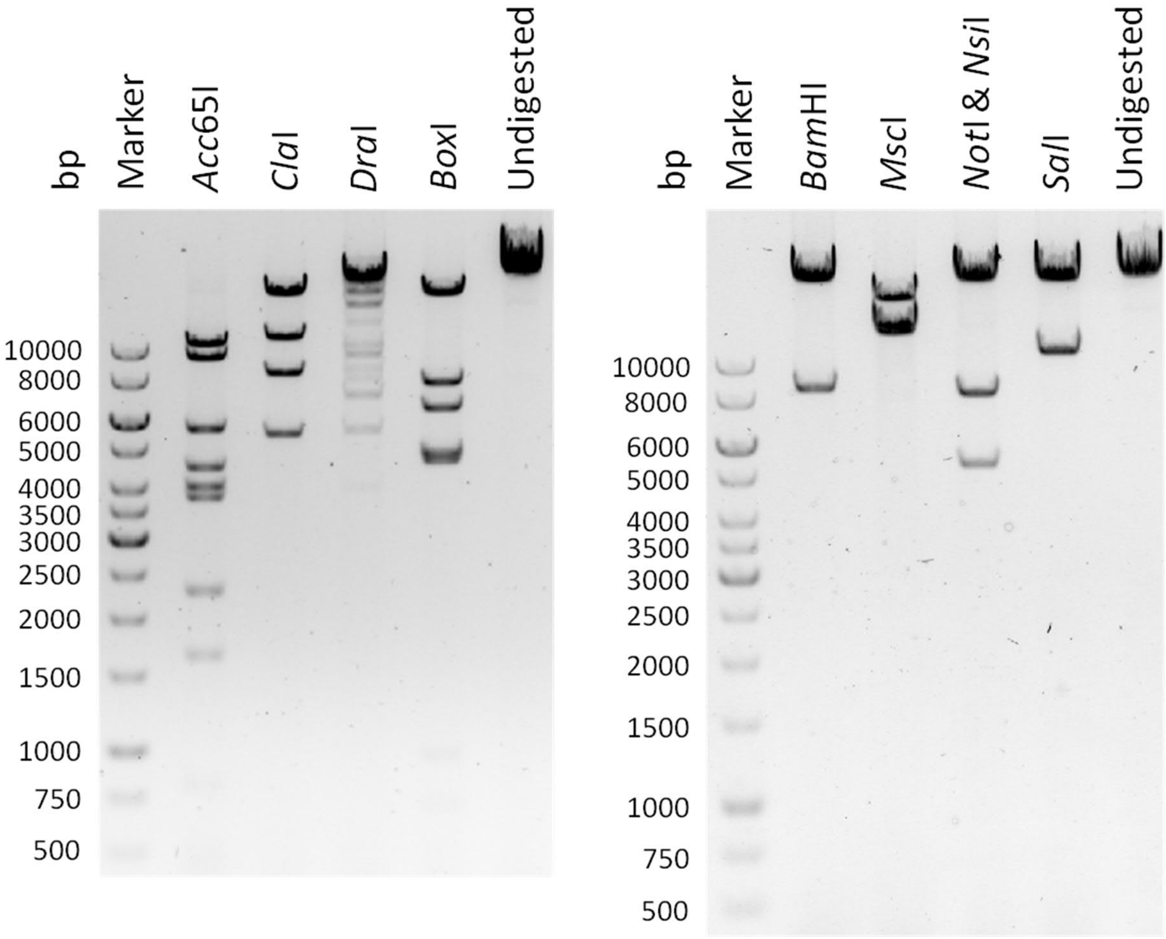

Figure S1. Restriction enzyme digestion analysis of fHe-Kpn01 DNA. The DNA digested with restriction endonucleases Acc65I, BamHI, BoxI, ClaI, DraI, MscI, NotI, NsiI, and SalI were loaded to the gel as indicated, along with undigested phage DNA and 1 kb DNA ladder (Marker).

>The 500-bp *fliC* fragment of *Y.enterocolitica* serotype O:3 strain

TCAACCATCACCAACCTGAAtaacacagtgaacaacctgacctcagcccgtagccgtattcaggatgcggattact
caactgaagtgtccaacatgagccgtgcacagattctgcaacaagctggtacttctgttctggctcaggctaacca
ggttccacaaactgtattgtctctgctgcgttaatattattcagctgattcttgcaagcctccatattatggggc
tttttttatttcagcgcagcggaatgatacgccggatatacagctaaaggtttggatgagcctgccgataataaaa
aagacggtgattgaagccgtgggcaaataagcctgaaccttagccgaatcatatttaaaggaatacctactatggc
ggtcattaacactaacagtttgtctctgctgactcagaacaacctgaataaatcccagtcttctttaggcaccgcc
attgagcgtttgtcttccggtctgcgtatcaacagcgcaaaaga

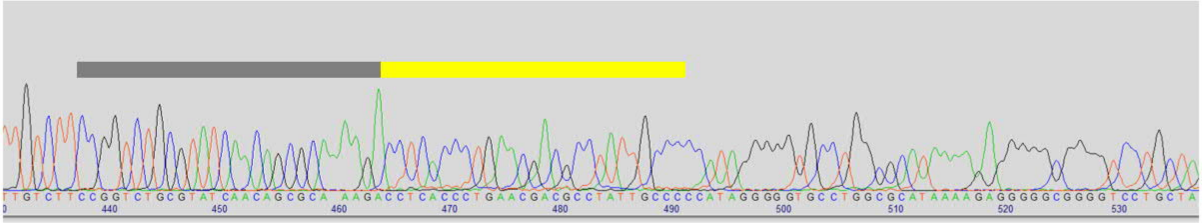

>fHe-Kpn01 Final, 43329 bp: rearranged based on PhageTerm prediction with 222 bp direct terminal repeats

CCTCACCCTGAACGACGCCTATTGCCCCCATAGGGGGTGCCTGGCGCATAAAAGAGGGGGCGGGGTCCTGCTA
GTGAGTGGCTAGTGCGCTGGGGCTGCACCTGCACGCCTCAGTGCGCTTCTATTACGCGCTAGGGCTATCGCTA
GTGCTACCCTATGCCTATCACTGTGCGTTCACTAGAGCGCCTCCTGTGCACTCTATGGGGCGCTAGGTGGGCG
CTCCGTCTTACCTATTTTGCGCACCTAGTGTGTCACCTAGTGGGGCCAGTAGCAGGCCTCTAGTGCCTGCCTA
GGGGGCCAGCTAGTGCGCTGTAGTACAGCACAGTGCTATCCCTAGTGCGCACTAGTCTATCGCTAGTGCTTTA

Figure S2. Determination of the physical ends of the fHe-Kpn01 genome. A 500 bp fliC fragment of Yersinia enterocolitica O:3 was ligated with the fHe-Kpn01 genome, the ligation junctions were amplified by PCR and sequenced. Shown are the sequences of the fliC fragment at the top and the left end of the fHe-Kpn01 genome at the bottom of the figure, and the Sanger sequencing read graph in the middle. The grey bar indicates sequence graph of the 3'-end of the fliC fragment, and the yellow bar the 5'-end graph of the fHe-Kpn01 genome.

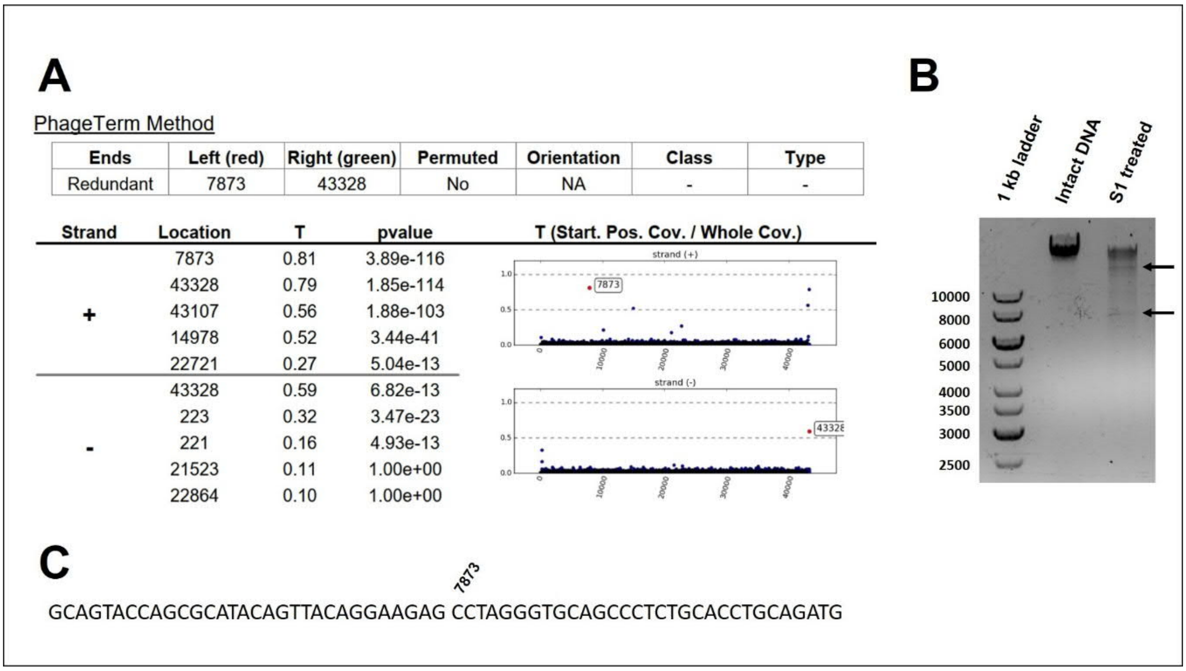

Figure S3. Detection of nicks in the genome of fHe-Kpn01. PhageTerm output (according to the PhageTerm method) showing major peaks in the ends of the genome on both positive and negative strands, and an additional major peak (marked 7873) in the positive strand (A). An agarose electrophoresis gel of genomic DNA of fHe-Kpn01 in intact form and digested with S1 nuclease to convert the putative nick into a double-strand break. In the S1-treated genomic sample two new DNA-fragments are evident (black arrows). A high molecular weight band is visible high up in the lane, and a weaker band, just below the 8 kb standard (B). These data together strongly support the presence of a nick at nucleotide position 7873 of the positive strand of the genome (C).

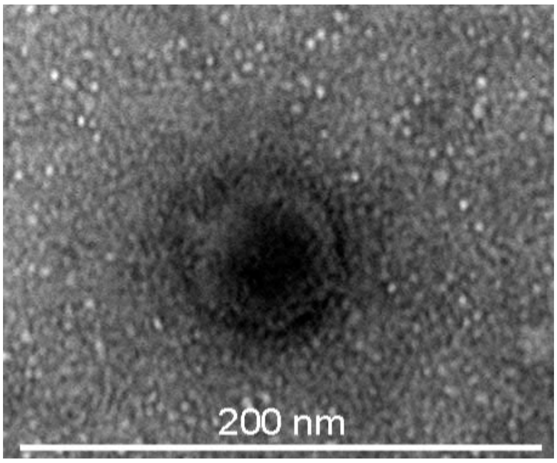

Figure S4. Transmission electron micrograph of a negatively stained fHe-Kpn01 particle.

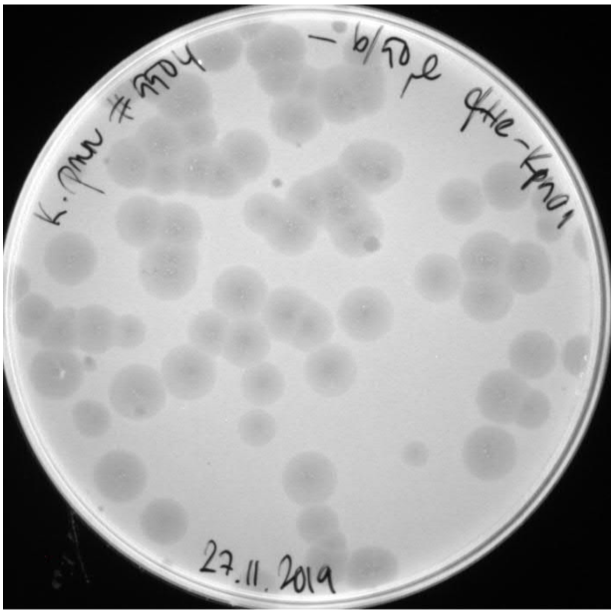

Figure S5. Plaque morphology of K. pneumoniae #5504 infected by fHe-Kpn01.

```
Gp57       MALSELVKAKSPVLLERQYLSSRIEAGALADTAIVAGLNENPGSILVVDIPVQVNSVVMP  60
SU503_53   MALSELVKAKSPVLLERQYLSSRIEAGALADTAIVAGLNENPGSILVVDIPVQVNSVVMP  60

Gp57       DNSILILAAQVTHRASASGHMLIPGSACTILGLGDAGGLNGNASNNPTQWRAVSCVNKSN  120
SU503_53   DNSTLILAAQVTHRASASGHMLIPGSACTILGLGDAGGLNGNAGNNPTQWRAVSCVNKSN  120

Gp57       VVLEDFFITNTKSAAAYFKGCTDTRTHKLRISNVVGTGEYAGGIYVTNCIRHKSSYNLIM  180
SU503_53   VVLEDFFITNTKSAAAYFEGCTDTRTHKLRISNVVGTGEYAGGIYVTNCIRHKSSYNLIM  180

Gp57       DVNTNGIKFRADTLGLTYGCSSDHDTIYRAGFIGIANGKCQNHKVTNWYAVDCVDNGVDM  240
SU503_53   DVNTNGIKFRADTLGLTYGCSSDHDSIYRAGFIGIANGKCQNHKVTNWYAVDCVDNGVDM  240

Gp57       NGCYDAVFDGGTSLRCQDGMYIGENNIDLCSVRNSTAIDCKRAGIGSMGSLTNCTMLDNF  300
SU503_53   NGCYDTVFDGGTSLRCQDGMYIGENNIDLCSVRNSAAIDCKRAGIGSMGSLTNCTMLDNF  300

Gp57       IDRCGSGIYASGYVGLSIRGNIIRNSSKKTYTDNETGQVKVSTGHGIGIQANLSAAHKTD  360
SU503_53   IDRCGSGIYASGYVGLSIRGNIIRNSSKKTYTDNETGMVKVSTGSGIDIQANLSAAYKTD  360

Gp57       IISNSFMSNAGYDVHWGVGTVADARMLDNDFVNTFGDGKVYLGSTFTNYQSKDNRGYRNE  420
SU503_53   IISNSFMSNAGYDVNWGVGTVADARMLDNDFVNTFGDGKVYLGSAFTNYQSKDNRGYLNE  420

Gp57       ATIVLNLTGNGTTVRFPVNLPFTAANTNYTVESVVPDWTSTYRVLLVDQTTTGFSLEFGT  480
SU503_53   ATIVLNLTGDGTTVRFQVNLPFKAANTNYTVESVVPDWTSTYRVLLVSQTTTGFLLEFGT  480

Gp57       APPAGSTRRVVVRVTGLVQA  500
SU503_53   APPAGSTRRVVVRVTGLVQA  500
```

Figure S6. Alignment of the receptor binding proteins of the fHe-Kpn01 and vB_KpnP_SU503 phages. The amino acid sequences of Gp57 (from fHe-Kpn01) and SU503_53 (from vB_KpnP_SU502) are aligned and the differences in residues are highlighted in grey.

viruses MDPI

Table S1. Bacterial strains used to determine the host range of fHe-Kpn01. The *Klebsiella pneumoniae* strains (human isolates) were mostly provided by the hospital district of Helsinki and Uusimaa laboratories (HUSLAB). Storage numbers in the Skurnik laboratory collection are shown. NK: not known; NA: not applicable. Strains that produce extended-spectrum beta-lactamase (ESBL) or carbapenemase (CPE) are indicated. If known, the capsule type and O-serotype are indicated. Strains that were infected by fHe-Kpn1 are marked with ++, the strain infected at low efficiency with +/–, and strains not infected with –.The sensitivity of the strains indicated in bold to fHe-Kpn01 was confirmed by determining infection growth curves using the Bioscreen C incubator.

Storage number	Species	Human origin	Resistance and other properties	Reference	Sensitivity to fHe-Kpn01
808	K. pneumoniae	NK		Turku University Hospital	–
2689	E. coli DH10B	NA		Invitrogen	–
5504	K. pneumoniae	urine	ESBL, Capsule type KL62, O-serotype O1v	HUSLAB	++
5505	K. pneumoniae	urine	ESBL	HUSLAB	–
5518	K. pneumoniae	blood		HUSLAB	–
5529	K. pneumoniae	blood		HUSLAB	+/–
5533	K. pneumoniae	blood		HUSLAB	–
5536	K. pneumoniae	blood		HUSLAB	–
5540	K. pneumoniae	blood		HUSLAB	–
5546	K. pneumoniae	blood		HUSLAB	–
5547	K. pneumoniae	blood		HUSLAB	–
5563	K. pneumoniae	feces	CPE	HUSLAB	–
5640	K. pneumoniae	urine		HUSLAB	–
5641	K. pneumoniae	urine		HUSLAB	–
5642	K. pneumoniae	urine		HUSLAB	–
5643	K. pneumoniae	urine		HUSLAB	–

5644	K. pneumoniae	urine		HUSLAB	–
5645	K. pneumoniae	urine		HUSLAB	–
5646	K. pneumoniae	urine		HUSLAB	–
5647	K. pneumoniae	tracheal mucus		HUSLAB	–
5648	K. pneumoniae	surgical wound secretion		HUSLAB	–
5649	K. pneumoniae	urine	ESBL	HUSLAB	–
5650	K. pneumoniae	urine		HUSLAB	–
5651	K. pneumoniae	feces	ESBL	HUSLAB	–
5653	K. pneumoniae	wound		HUSLAB	–
5654	K. pneumoniae	urine		HUSLAB	–
5655	K. pneumoniae	urine		HUSLAB	–
5656	K. pneumoniae	urine		HUSLAB	–
5748	K. pneumoniae	NK	ESBL	HUSLAB	–
5749	K. pneumoniae	urine		HUSLAB	–
5750	K. pneumoniae	gingival pouch		HUSLAB	–
5751	K. pneumoniae	finger burn wound secretion		HUSLAB	–
5752	K. pneumoniae	NK	ESBL	HUSLAB	–
5771	K. pneumoniae	bronchial secretion		HUSLAB	–
5772	K. pneumoniae	urine		HUSLAB	–
5773	K. pneumoniae	urine		HUSLAB	–
5774	K. pneumoniae	urine		HUSLAB	–
5775	K. pneumoniae	urine		HUSLAB	–
5776	K. pneumoniae	urine		HUSLAB	–

5777	K. pneumoniae	feces	ESBL	HUSLAB	–
5778	K. pneumoniae	blood	EBSL	HUSLAB	–
5779	K. pneumoniae	urine, catheter		HUSLAB	–
5780	K. pneumoniae	neck wound	ESBL	HUSLAB	–
5781	K. pneumoniae	urine		HUSLAB	–
5782	K. pneumoniae	urine		HUSLAB	–
5783	K. pneumoniae	urine		HUSLAB	–
5784	K. pneumoniae	ankle biopsy		HUSLAB	–
5785	K. pneumoniae	thigh burn wound		HUSLAB	–
5786	K. pneumoniae	cervical secretion		HUSLAB	–
5787	K. pneumoniae	perianal abscess		HUSLAB	–
5788	K. pneumoniae	jaw fistula		HUSLAB	–
5789	K. pneumoniae	urine		HUSLAB	–
5790	K. pneumoniae	urine		HUSLAB	–
6037	K. pneumoniae		Capsule type KL2, O-serotype O1	ATCC 43816	–
6038	K. pneumoniae		Capsule type KL107	ATCC 700721, MGH78578	–
6039	K. pneumoniae		Capsule KL1, O-serotype O1	NTUH-K2044	–
6069	K. pneumoniae	feces	ESBL	HUSLAB	–
6322	K. pneumoniae		ESBL, Capsule type KL2	KP1, ATCC10031, [1]	–
6323	K. pneumoniae	NK	ESBL	KP2, [1]	–
6324	K. pneumoniae	NK	ESBL	KP4, [1]	–
6325	K. pneumoniae	NK	ESBL	KP5, [1]	–
6326	K. pneumoniae	NK	ESBL, Capsule type KL62, O-serotype O1v	KP6, [1]	++
6470	K. pneumoniae	NK		HUSLAB	–
6596	K. pneumoniae	NK		HUSLAB	–

6738	K. pneumoniae	NK	Capsule type KL49	HUSLAB	–
6739	K. oxytoca	NK		HUSLAB	–
6740	K. pneumoniae	nephritis	Capsule type KL64	HUSLAB	–
6815	K. pneumoniae	nasal swab		HUSLAB	–
6828	K. pneumoniae	sinus secretion		HUSLAB	–
6884	K. pneumoniae	feces		HUSLAB	–
6896	K. pneumoniae	nasal swab		HUSLAB	–
6897	K. pneumoniae	feces		HUSLAB	–
6905	K. pneumoniae	nasal swab		HUSLAB	–
6911	K. pneumoniae	nasal swab		HUSLAB	–

Reference

1. Mattila, S.; Ruotsalainen, P.; Jalasvuori, M. On-demand isolation of bacteriophages against drug-resistant bacteria for personalized phage therapy. Front Microbiol 2015, 6, 1271.

Table S2. The predicted gene products of fHe-Kpn01. The genes, gene location, gene size, and protein length are listed. LC-MS/MS analysis was performed to identify phage particle associated proteins (PPAPs). The best HHpred hits (with a probability above 50% and E-value below 1) are included. A BLASTP search against the non-redundant protein sequences database (release 2.9.0 from April 1, 2019) was performed for every predicted gene product and the two results with the lowest E-values were recorded including query coverage, amino acid identity, and the accession numbers of the closest homologs.

Gene	Start position	End position	Gene size (bp)	Protein size (aa)	PPAP	The best HHpred hits (Probability percentage/E-value)	The best BLASTP hits	BLASTP query coverage (%)	BLASTP score (E-value)	BLASTP amino acid identity (%)	BLASTP accession number
g02	1438	1653	216	71	no	No significant hits	hypothetical protein F19_02 [Klebsiella phage F19]	100	1e-41	97.18	YP_009006022.1
							hypothetical protein phiKpS2_1 [Klebsiella phage phiKpS2]	100	2e-41	95.77	AWK23995.1
g03	1732	2316	585	194	no	No significant hits	hypothetical protein kpv41_02 [Klebsiella phage KpV41]	100	8e-135	97.42	YP_009188744.1
							hypothetical protein AltoGao_2 [Klebsiella phage AltoGao]	100	2e-132	95.36	ASV44895.1
g04	2313	2444	132	43	no	No significant hits	hypothetical protein SU552A_03 [Klebsiella phage vB_KpnP_SU552A]	100	3e-21	90.70	YP_009204792.1
							hypothetical protein kpv74_03 [Klebsiella phage vB_KpnP_KpV74]	100	5e-21	90.70	APZ82715.1
g05	2491	2760	270	89	no	No significant hits	hypothetical protein SU503_04 [Klebsiella phage vB_KpnP_SU503]	91	8e-53	98.77	YP_009199888.1
							hypothetical protein [Klebsiella phage Kp2]	89	3e-37	77.50	YP_009188315.1

g06	2819	3040	222	73	no	No significant hits	hypothetical protein KPRIO2015_4 [Klebsiella phage KP-Rio/2015]	100	3e-41	86.30	AOT23843.1
							hypothetical protein kpv74_04 [Klebsiella phage vB_KpnP_KpV74]	100	1e-40	84.93	APZ82716.1
g07	3033	3287	255	84	no	No significant hits	hypothetical protein AltoGao_5 [Klebsiella phage AltoGao]	100	6e-50	92.13	ASV44898.1
							hypothetical protein kpv41_05 [Klebsiella phage KpV41]	100	7e-48	89.66	YP_009188747.1
g08	3303	3542	240	79	no	No significant hits	hypothetical protein AltoGao_6 [Klebsiella phage AltoGao]	100	2e-49	97.47	ASV44899.1
							hypothetical protein BO1E_0006 [Klebsiella phage phiBO1E]	98	7e-42	85.90	AIT13575.1
g09	3539	3724	186	61	no	No significant hits	hypothetical protein AltoGao_7 [Klebsiella phage AltoGao]	100	7e-35	93.44	ASV44900.1
							hypothetical protein SU503_07 [Klebsiella phage vB_KpnP_SU503]	95	2e-34	98.28	YP_009199891.1
g10	3721	3930	210	69	no	No significant hits	hypothetical protein SU503_08 [Klebsiella phage vB_KpnP_SU503]	100	1e-43	98.55	YP_009199892.1
							hypothetical protein [Klebsiella phage Kp2]	100	5e-22	65.22	YP_009188319.1
g11	3918	4262	345	114	no	No significant hits	hypothetical protein [Klebsiella phage KP34]	100	6e-66	85.09	YP_003347659.1
							hypothetical protein SU503_09 [Klebsiella phage vB_KpnP_SU503]	100	8e-66	96.49	YP_009199893.1

g12	4312	5982	1671	556	yes	No significant hits	hypothetical protein SU503_10 [Klebsiella phage vB_KpnP_SU503]	100	0.0	95.32	YP_009199894.1
							hypothetical protein kpv48_08 [Klebsiella phage vB_KpnP_KpV48]	98	0.0	86.31	AOZ65216.1
g13	5982	7028	1047	348	yes	4H2K_B, Succinyl-diaminopimelate desuccinylase {Haemophilus influenzae} (99.78%/5.7*10^{-17})	putative peptidase [Klebsiella phage KP34]	100	0.0	96.84	YP_003347662.1
							hypothetical protein [Klebsiella phage Kp2]	100	0.0	96.55	YP_009188322.1
g14	7031	7498	468	155	yes	2RH2_A, Dihydrofolate reductase type 2 {Escherichia coli} (92.42%/0.31)	hypothetical protein SU503_12 [Klebsiella phage vB_KpnP_SU503]	98	9e-99	90.20	YP_009199896.1
							hypothetical protein AltoGao_12 [Klebsiella phage AltoGao]	98	5e-81	75.16	ASV44905.1
g15	7495	7683	189	62	no	No significant hits	hypothetical protein kpv41_13 [Klebsiella phage KpV41]	100	5e-28	77.42	YP_009188755.1
							hypothetical protein phiKpS2_12 [Klebsiella phage phiKpS2]	79	9e-12	63.27	AWK24006.1
g16	7664	8455	792	263	no	2AU3_A, DNA primase {Aquifex aeolicus} (99.83%/9.8*10^{-19})	DNA primase/helicase [Klebsiella phage KPV811]	100	0.0	97.34	APD20690.1
							putative DNA primase [Klebsiella phage KP34]	98	0.0	99.23	YP_003347665.1
g17	8446	9729	1284	427	no	4NMN_B, Replicative DNA helicase {Aquifex aeolicus} (99.97%/3.2*10^{-26})	putative DNA helicase [Klebsiella phage KpV71]	99	0.0	97.88	YP_009302721.1
							putative DNA helicase [Klebsiella phage vB_KpnP_SU552A]	99	0.0	97.88	YP_009204805.1
g18	9841	9984	144	47	no	No significant hits	No significant similarity found				

g19	10044	10199	156	51	no	No significant hits	hypothetical protein SU503_16 [Klebsiella phage vB_KpnP_SU503]	100	3e-28	100.00	YP_009199900.1
							hypothetical protein kpv48_16 [Klebsiella phage vB_KpnP_KpV48]	100	3e-27	96.08	AOZ65224.1
g20	10196	10552	357	118	no	No significant hits	hypothetical protein SU503_17 [Klebsiella phage vB_KpnP_SU503]	100	1e-83	99.15	YP_009199901.1
							hypothetical protein BO1E_0021 [Klebsiella phage phiBO1E]	100	5e-47	55.93	AIT13590.1
g21	10542	12896	777	258	no	2KFN_A, Klenow fragment of DNA polymerase {Escherichia coli} (100%/1.6*10^{-69})	DNA polymerase I [Klebsiella phage myPSH1235]	100	0.0	98.45	AVP40054.1
							DNA polymerase [Klebsiella phage KP34]	100	0.0	97.67	YP_003347671.1
g22	12893	13441	549	182	no	4WBY_A, RNA Nucleotidyltransferase {Aquifex aeolicus} (99.83%/3.3*10^{-19})	hypothetical protein SU503_19 [Klebsiella phage vB_KpnP_SU503]	100	2e-131	98.90	YP_009199903.1
							putative nucleotidyltransferase [Proteus phage PM16]	83	7e-22	38.96	YP_009147851.1
g23	13431	13652	222	73	no	No significant hits	hypothetical protein SU503_20 [Klebsiella phage vB_KpnP_SU503]	100	7e-46	98.63	YP_009199904.1
							hypothetical protein BO1E_0023 [Klebsiella phage phiBO1E]	100	6e-42	93.15	AIT13592.1
g24	13815	14792	978	325	yes	3RQZ_A, Metallophosphoesterase {Sphaerobacter thermophilus} (99.59%/4.1*10^{-14})	hypothetical protein [Shigella phage SFN6B]	100	0.0	94.15	AVD98972.1
							hypothetical protein [Klebsiella phage KP34]	99	0.0	87.43	YP_003347674.1

g25	14806	14970	165	54	no	No significant hits	hypothetical protein kpv475_20 [Klebsiella phage KpV475]	100	1e-24	96.30	YP_009280689.1
							hypothetical protein F19_18 [Klebsiella phage F19]	100	8e-23	88.89	YP_009006039.1
g26	15022	15855	834	277	yes	No significant hits	hypothetical protein kpv475_21 [Klebsiella phage KpV475]	100	0.0	98.92	YP_009280690.1
							large tegument protein [Klebsiella phage vB_KpnP_SU503]	100	0.0	97.83	YP_009199907.1
g27	15908	16162	255	84	yes	No significant hits	hypothetical protein SU503_24 [Klebsiella phage vB_KpnP_SU503]	100	5e-51	100.00	YP_009199908.1
							hypothetical protein kpv41_24 [Klebsiella phage KpV41]	100	4e-50	97.62	YP_009188766.1
g28	16163	16441	279	92	yes	No significant hits	hypothetical protein SU503_25 [Klebsiella phage vB_KpnP_SU503]	100	5e-60	98.91	YP_009199909.1
							hypothetical protein kpv475_23 [Klebsiella phage KpV475]	100	6e-60	97.83	YP_009280692.1
g29	16441	16812	372	123	no	No significant hits	hypothetical protein kpv475_24 [Klebsiella phage KpV475]	99	8e-77	94.26	YP_009280693.1
							hypothetical protein kpv74_25 [Klebsiella phage vB_KpnP_KpV74]	99	1e-76	93.44	APZ82737.1
g30	16815	16976	162	53	no	2KSD_A, Aerobic respiration control sensor protein {Escherichia coli} (91.76%/0.88)	hypothetical protein SU503_27 [Klebsiella phage vB_KpnP_SU503]	100	1e-29	100.00	YP_009199911.1
							hypothetical protein AltoGao_26 [Klebsiella phage AltoGao]	100	3e-18	66.04	ASV44919.1

g31	16976	17944	969	322	yes	5HML_B, Exodeoxyribonuclease {Escherichia phage T5} (99.97%/1.2*10^{-30})	putative 5'-3' exonuclease [Klebsiella phage vB_KpnP_SU503]	100	0.0	100.00	YP_009199912.1
							hypothetical protein [Klebsiella phage KPV811]	100	0.0	98.76	APD20680.1
g32	17901	18104	204	67	no	No significant hits	hypothetical protein SU503_29 [Klebsiella phage vB_KpnP_SU503]	100	1e-40	100.00	YP_009199913.1
							hypothetical protein [Klebsiella phage Kp2]	100	2e-40	98.51	YP_009188343.1
g33	18095	18517	423	140	yes	1E7L_B, Recombination endonuclease VII {Bacteriophage T4} (99.75%/4.3*10^{-18})	endonuclease [Klebsiella phage Kp2]	100	5e-97	100.00	YP_009188344.1
							putative DNA endonuclease VII [Klebsiella phage myPSH1235]	100	8e-97	99.29	AVP40068.1
g34	18514	18972	459	152	yes	No significant hits	hypothetical protein SU503_31 [Klebsiella phage vB_KpnP_SU503]	100	5e-112	100.00	YP_009199915.1
							hypothetical protein [Klebsiella phage Kp2]	100	2e-89	82.89	YP_009188345.1
g35	18962	19078	117	38	no	No significant hits	hypothetical protein [Klebsiella phage Kp2]	100	9e-20	94.74	YP_009188346.1
							hypothetical protein kpv48_35 [Klebsiella phage vB_KpnP_KpV48]	100	7e-19	94.74	AOZ65243.1
g36	19117	21585	2469	822	yes	1MSW_D, DNA-directed RNA polymerase {Enterobacteria phage T7} (100%/3.6*10^{-147})	DNA-dependent RNA polymerase [Klebsiella phage vB_KpnP_SU503]	100	0.0	99.51	YP_009199916.1
							RNA polymerase [Klebsiella phage Kp2]	99	0.0	99.03	YP_009188347.1

g37	21593	22069	477	158	no	1U3E_M, HNH catalytic motif {Bacillus phage SPO1} (99.5%/1.4*10^{-13})	endonuclease of the HNH familly [Klebsiella phage vB_KpnP_SU503]	100	1e-114	100.00	YP_009199917.1
							putative HNH endonuclease [Klebsiella phage NTUH-K2044-K1-1]	100	4e-111	97.47	YP_009098369.1
g38	22093	22533	441	146	no	3I3G_A, N-acetyltransferase {Trypanosoma brucei} (97.7%/0.0044)	hypothetical protein SU503_34 [Klebsiella phage vB_KpnP_SU503]	100	2e-103	100.00	YP_009199918.1
							hypothetical protein [Klebsiella phage Kp2]	100	5e-103	99.32	YP_009188348.1
g39	22530	22793	264	87	yes	No significant hits	hypothetical protein [Klebsiella phage KP34]	100	3e-50	98.85	YP_003347631.1
							hypothetical protein AltoGao_35 [Klebsiella phage AltoGao]	100	2e-49	97.70	ASV44928.1
g40	22803	24398	1596	531	yes	6R21_C, viral complex, DNA ejection {Enterobacteria phage T7} (100%/2*10^{-50})	head-tail connector protein [Klebsiella phage vB_KpnP_SU503]	100	0.0	99.25	YP_009199920.1
							head-tail connector protein [Klebsiella phage vB_KpnP_SU552A]	100	0.0	98.49	YP_009204826.1
g41	24413	25255	843	280	yes	No significant hits	putative scaffolding protein [Klebsiella phage KpV41]	100	0.0	98.93	YP_009188780.1
							putative scaffolding protein [Klebsiella phage vB_KpnP_SU552A]	100	0.0	98.57	YP_009204827.1
g42	25281	26300	1020	339	yes	2XD8_C, T7-like capsid protein {Prochlorococcus phage P-SSP7} (99.95%/1.3*10^{-25})	capsid protein [Klebsiella phage vB_KpnP_SU503]	100	0.0	97.64	YP_009199922.1
							putative capsid protein [Klebsiella phage vB_KpnP_KpV48]	100	0.0	97.94	AOZ65249.1
g43	26312	26494	183	60	yes	No significant hits	hypothetical protein [Klebsiella phage KP34]	100	1e-29	93.33	YP_003347637.1
							hypothetical protein AltoGao_39 [Klebsiella phage AltoGao]	100	9e-29	91.67	ASV44932.1

g44	26583	27143	561	186	yes	3J4B_F, Tail tubular protein A {Enterobacteria phage T7} (100%/9.8*10-39)	tail tubular protein A [Klebsiella phage vB_KpnP_SU503]	100	9e-133	98.92	YP_009199924.1
							tail fibers protein [Klebsiella phage KPV811]	100	2e-131	96.24	APD20670.1
g45	27154	29529	2376	791	yes	6R21_E, viral complex, DNA ejection {Enterobacteria phage T7} (100%/1.7*10-101)	putative tail tubular protein B [Klebsiella phage vB_KpnP_SU503]	100	0.0	99.49	YP_009199925.1
							putative tail tubular protein B [Klebsiella phage KpV41]	98	0.0	98.98	YP_009188784.1
g46	29531	30118	588	195	yes	No significant hits	putative internal virion protein B [Klebsiella phage F19]	100	4e-136	99.49	YP_009006062.1
							putative internal virion protein B [Klebsiella phage vB_KpnP_SU552A]	100	2e-135	98.97	YP_009204832.1
g47	30136	32820	2685	894	yes	6D9M_A, Endolysin, Response receiver sensor diguanylate cyclase {Enterobacteria phage T4} (97.02%/0.0048)	hypothetical protein [Klebsiella phage KP34]	100	0.0	96.09	YP_003347641.1
							hypothetical protein kpv41_44 [Klebsiella phage KpV41]	100	0.0	95.41	YP_009188786.1
g48	32871	36569	3699	1232	yes	No significant hits	putative internal core protein [Klebsiella phage vB_KpnP_SU503]	100	0.0	99.27	YP_009199928.1
							methyl-accepting chemotaxis protein I [Klebsiella phage Kp2]	100	0.0	98.46	YP_009188358.1
g49	36571	37494	924	307	yes	5JS4_A, phiAB6 tailspike {unidentified phage} (97.93%/0.00013)	putative tail fiber protein [Klebsiella phage vB_KpnP_SU503]	100	0.0	99.67	YP_009199929.1
							putative tail fiber protein [Klebsiella phage F19]	100	0.0	94.46	YP_009006065.2

g50	37507	37809	303	100	yes	No significant hits	hypothetical protein [Klebsiella phage Kp2]	100	2e-62	99.00	YP_009188360.1
							putative DNA maturase A [Klebsiella phage vB_KpnP_SU503]	100	3e-62	99.00	YP_009199930.1
g51	37809	39665	1857	618	yes	4BIJ_C, DNA maturase B {Enterobacteria phage T7} (100%/$3.6*10^{-35}$)	putative DNA maturase B [Klebsiella phage vB_KpnP_SU503]	100	0.0	99.51	YP_009199931.1
							putative DNA maturase B [Klebsiella phage KpV475]	100	0.0	99.35	YP_009280714.1
g52	39665	40039	375	124	yes	No significant hits	hypothetical protein kpv71_48 [Klebsiella phage KpV71]	99	5e-82	100.00	YP_009302752.1
							hypothetical protein [Klebsiella phage KP34]	99	1e-81	99.19	YP_003347646.1
g53	40051	40233	183	60	yes	No significant hits	hypothetical protein SU503_49 [Klebsiella phage vB_KpnP_SU503]	100	3e-31	98.33	YP_009199933.1
							hypothetical protein kpv41_51 [Klebsiella phage KpV41]	100	1e-30	96.67	YP_009188793.1
g54	40233	40637	405	134	yes	3ZBI_R, TRAF protein {Escherichia coli} (92.79%/0.085)	hypothetical protein [Klebsiella phage KP34]	100	7e-88	99.25	YP_003347648.1
							putative spanin protein [Klebsiella phage myPSH1235]	100	1e-87	99.25	AVP40087.1
g55	40630	40881	252	83	no	2M20_B, Epidermal growth factor receptor {Homo sapiens} (91.88%/1)	putative holin [Klebsiella phage vB_KpnP_SU503]	100	9e-53	100.00	YP_009199935.1
							hypothetical protein F19_49 [Klebsiella phage F19]	100	2e-52	98.80	YP_009006072.1
g56	40865	41473	609	202	yes	6ET6_A, Lysozyme {Acinetobacter baumannii} (99.85%/$3.8*10^{-20}$)	putative endolysin [Klebsiella phage vB_KpnP_SU503]	100	1e-146	100.00	YP_009199936.1
							endolysin [Klebsiella phage phiKpS2]	98	1e-138	93.97	AWK24046.1

g57	41474	42976	1503	500	yes	3SUC_A, Preneck appendage protein {Bacillus phage phi29} (98.19%/0.0044)	putative pectate lyase SU503_53 [Klebsiella phage vB_KpnP_SU503]	100	0.0	96.40	YP_009199937.1
							hypothetical protein [Klebsiella pneumoniae]	91	3e-128	47.72	WP_101998518.1

viruses MDPI

Table S3. Primers used to amplify the HPUF genes from fHe-Kpn01. Sequences are presented in 5´to 3´direction and restriction sites (NcoI, NheI or NotI) are underlined. Size of the gene in basepairs (bp) is given from start to stop codon.

Gene	Forward primer	Reverse primer	Size (bp)
g02	GCAGCGGCCGCATGAAGTACAAAGATAAGTTGAA	GGTCCATGGTTAGTGTCCGAACTGTCC	216
g03	GCAGCGGCCGCATGACTAACTCCACCAATTCA	GGTCCATGGTCATGCCGCTGCCTTAAA	585
g04	GCAGCGGCCGCATGATTTATAAAGCTCCGGC	GGTCCATGGCTAGAGATACTCACCCAATCC	132
g05	GCAGCGGCCGCATGCCAACTAAAAGGAACAA	GGTCCATGGCTACTGGACCGGTTGGAA	270
g06	GCAGCGGCCGCATGAAAGCAATACTGGTTTATC	GGTCCATGGTTAACCATAAAGTTGTCTCCA	222
g07	GCAGCGGCCGCATGGTTAACGTATTCAATATCAT	GGTCCATGGTTAAATTTCGTTGTTGCTCTTA	255
g08	GCAGCGGCCGCATGAAAGTCTTAAAGTTCATTCGT	GGTCCATGGTCATGCCCCGCCTCC	240
g09	GCAGCGGCCGCATGAAGTCTTACGGAAAGAATC	GGTCCATGGTCATACTATGCCCCGCAC	186
g10	GCAGCGGCCGCATGATTAAGTACGATGTATACAAG	GGTCCATGGCTACTGTGAGCATAGGCTG	210
g11	GCAGCGGCCGCATGCTCACAGTAGACGAAA	GGTCCATGGTCAGCGCGCATACCAC	345
g15	GCAGCGGCCGCATGAAAGTAAGAGCGGTGA	GGTCCATGGCTAAGCCAGGGCTGGTC	189
g18	GCAGCGGCCGCATGTATAATAAAATATGTTCTGGT ATG	GGTCCATGGTTATTCTTGTTTCTCAAACTGT AT	144
g19	GCAGCGGCCGCATGCTTAAACCAGAAGATATCA	GGTCCATGGTCATTTCCACTTAGCCCT	156
g20	GCAGCGGCCGCATGAAAGCTAAGCACATCAA	GGTCCATGGCTAGTTGTCATTGTTTAGCCT	357
g22	GCAGCGGCCGCATGATTGACAGAGAAGAGATAC	GGTCCATGGTTAATATGCATCACGCACC	549
g23	GCAGCGGCCGCATGCATATTAAACCTGGCAG	GGTCCATGGTCACTCCGCTATAAGTTTGT	222
g25	GCAGCGGCCGCATGAAAATGGGAATCTGTTCTG	GGTCCATGGTTAGCGTATGGAGGCCAC	165
g29	GCAGCGGCCGCATGGACCAAGTACTGAACG	GGTCCATGGTTAGAGCTCCAATTCAGAGAG	372
g30	GCAGCGGCCGCATGCGTACTGCATTCGCTA	GGTCCATGGTTAAACCCCCGTGACCTTTTT	162
g32	GCAGCGGCCGCATGGCGAAGATGTCTGCG	GGTCCATGGTTAGGGCCATACCTCAATCT	204
g35	GCAGCGGCCGCTTGACGCCTAGCGAATGG	GGTCCATGGCTACTGATTGCGACTTACCC	117
g38	GCAGCGGCCGCATGAAGTTAAAACACACTAGTAA	GGTCCATGGTCATTCTGACCTCACTAAATG	441

viruses MDPI

Table S4. Genome typing analysis of representative K. pneumoniae strains carried out with the Kleborate tool. ^, exact amino acid match but with 1 or more nucleotide differences; ?, partial match to the reported allele; *, no exact match but closest nucleotide match; +/-, with/without match.

strain	species	species_match	contig_count	N50	largest_contig	ambiguous_bases	ST
Kpn_5504_scaffolds	Klebsiella pneumoniae	strong	69	534519	803049	yes	ST48
Kpn_6326_scaffolds	Klebsiella pneumoniae	strong	104	296483	762655	yes	ST48
Kpn_ATCC_10031	Klebsiella pneumoniae	strong	1	5278269	5278269	no	ST86
Kpn_ATCC_700721	Klebsiella pneumoniae	strong	19	5303843	5303843	no	ST38
Kpn_ATCC_43816	Klebsiella pneumoniae	strong	10	5175574	5175574	no	ST493
strain	virulence_score	resistance_score	num_resistance_classes	num_resistance_genes	Yersiniabactin	YbST	Colibactin
Kpn_5504_scaffolds	0	1	5	5	-	0	-
Kpn_6326_scaffolds	1	1	7	10	ybt 10; ICEKp4	26-2LV	-
Kpn_ATCC_10031	0	0	1	0	-	0	-
Kpn_ATCC_700721	0	0	6	14	-	0	-
Kpn_ATCC_43816	1	0	1	0	ybt 2; ICEKp1	321	-
strain	CbST	Aerobactin	AbST	Salmochelin	SmST	rmpA	rmpA2
Kpn_5504_scaffolds	0	-	0	-	0	-	-
Kpn_6326_scaffolds	0	-	0	-	0	-	-
Kpn_ATCC_10031	0	-	0	-	0	rmpA_2(KpVP-1)	-
Kpn_ATCC_700721	0	-	0	-	0	-	-
Kpn_ATCC_43816	0	-	0	iro 3	21	rmpA_11*(ICEKp1)	-
strain	wzi	K_locus	K_locus_problems	K_locus_confidence	K_locus_identity	K_locus_missing_genes	O_locus
Kpn_5504_scaffolds	wzi62	KL62	-	High	99.72%	KL62_12	O1v1
Kpn_6326_scaffolds	wzi62	KL62	?-	Good	99.72%	KL62_12	O1v1
Kpn_ATCC_10031	wzi2	KL2	-	High	99.94%	KL2_14_wcaJ	O2v1

Kpn_ATCC_700721	wzi50	KL107	?-+*	None	86.04%	KL107_05_wzb,KL107_06_wzc,KL107_07_wbaP,KL107_08,KL107_09,KL107_10	OL101
Kpn_ATCC_43816	wzi2	KL2	none	Very high	99.72%		O1v1
strain	O_locus_problems	O_locus_confidence	O_locus_identity	O_locus_missing_genes	Chr_ST	gapA	infB
Kpn_5504_scaffolds	none	Very high	97.87%		ST48	2	5
Kpn_6326_scaffolds	none	Very high	97.86%		ST48	2	5
Kpn_ATCC_10031	-+	Good	98.45%	O1/O2v1_01_wzm	ST86	9	4
Kpn_ATCC_700721	*	High	94.91%		ST38	2	1
Kpn_ATCC_43816	none	Very high	99.81%		ST493	2	1
strain	mdh	pgi	phoE	rpoB	tonB	ybtS	ybtX
Kpn_5504_scaffolds	2	2	7	1	10	-	-
Kpn_6326_scaffolds	2	2	7	1	10	16	12*
Kpn_ATCC_10031	2	1	1	1	27	-	-
Kpn_ATCC_700721	2	1	2	2	2	-	-
Kpn_ATCC_43816	70	1	12	1	127	8	55
strain	ybtQ	ybtP	ybtA	irp2	irp1	ybtU	ybtT
Kpn_5504_scaffolds	-	-	-	-	-	-	-
Kpn_6326_scaffolds	4	3	3	35	50	3	10
Kpn_ATCC_10031	-	-	-	-	-	-	-
Kpn_ATCC_700721	-	-	-	-	-	-	-
Kpn_ATCC_43816	50	6	5	117	125	6	7
strain	ybtE	fyuA	clbA	clbB	clbC	clbD	clbE
Kpn_5504_scaffolds	-	-	-	-	-	-	-
Kpn_6326_scaffolds	9	2	-	-	-	-	-
Kpn_ATCC_10031	-	-	-	-	-	-	-

Kpn_ATCC_700721	-	-	-	-	-	-	-
Kpn_ATCC_43816	7	6	-	-	-	-	-
strain	clbF	clbG	clbH	clbI	clbL	clbM	clbN
Kpn_5504_scaffolds	-	-	-	-	-	-	-
Kpn_6326_scaffolds	-	-	-	-	-	-	-
Kpn_ATCC_10031	-	-	-	-	-	-	-
Kpn_ATCC_700721	-	-	-	-	-	-	-
Kpn_ATCC_43816	-	-	-	-	-	-	-
strain	clbO	clbP	clbQ	iucA	iucB	iucC	iucD
Kpn_5504_scaffolds	-	-	-	-	-	-	-
Kpn_6326_scaffolds	-	-	-	-	-	-	-
Kpn_ATCC_10031	-	-	-	-	-	-	-
Kpn_ATCC_700721	-	-	-	-	-	-	-
Kpn_ATCC_43816	-	-	-	-	-	-	-
strain	iutA	iroB	iroC	iroD	iroN	AGly	Col
Kpn_5504_scaffolds	-	-	-	-	-	-	-
Kpn_6326_scaffolds	-	-	-	-	-	StrA^;StrB	-
Kpn_ATCC_10031	-	-	-	-	-	-	MgrB-49%
Kpn_ATCC_700721	-	-	-	-	-	Aac6-Ib;AadA1-pm*?;AadB;Aph3''Ia;StrA;StrB	-
Kpn_ATCC_43816	-	6	38	10	5	-	-
strain	Fcyn	Flq	Gly	MLS	Ntmdz	Phe	Rif
Kpn_5504_scaffolds	-	GyrA-83Y	-	-	-	CatB4?	-
Kpn_6326_scaffolds	-	QnrB1?	-	MphA	-	-	-
Kpn_ATCC_10031	-	-	-	-	-	-	-

Kpn_ATCC_700721	-	GyrA-83Y	-	-	-	CatA1*;CmlA5	-
Kpn_ATCC_43816	-	-	-	-	-	-	-
strain	SuI	Tet	Tgc	Tmt	Omp	Bla	Bla_Carb
Kpn_5504_scaffolds	-	TetA	-	-	-	OXA-1;TEM-1D^	-
Kpn_6326_scaffolds	SulI;SulII	-	-	DfrA14;DfrA7	-	TEM-1D^	-
Kpn_ATCC_10031	-	-	-	-	-	SHV-187*	-
Kpn_ATCC_700721	SulI;SulII	TetD	-	-	-	OXA-9*;SHV-187*?;TEM-1D^;TEM-1D^	-
Kpn_ATCC_43816	-	-	-	-	-	-	-
strain	Bla_ESBL	Bla_ESBL_inhR	Bla_broad	Bla_broad_inhR			
Kpn_5504_scaffolds	CTX-M-15	-	SHV-1?	-			
Kpn_6326_scaffolds	CTX-M-15	-	SHV-11^	-			
Kpn_ATCC_10031	-	-	-	-			
Kpn_ATCC_700721	-	-	SHV-11^	-			
Kpn_ATCC_43816	-	-	-	SHV-26*			

viruses

MDPI

Table S5. CFU counts relative to the CFU of Gp178 in that batch of electroporations. Before calculating the CFU relative to the CFU of Gp178, the average CFU over triplicate plates was calculated. Every column indicates one batch of electroporations. The relative CFUs for Gp178 are relative to the average CFU of all electroporations of Gp178.

Gene	CFUs relative to Gp178 in that batch of electroporations. One column is one batch of electroporations.										Average	SD
RegB	0,104	0,131	0,064	0,006	0,011	0,009	0,036	0,085	0,108		0,062	0,045
Gp178	0,468	0,503	0,476	1,232	1,270	1,820	1,680	0,784	0,766		1,000	0,491
Gp2	0,725			0,600							0,662	0,062
Gp3	0,464			0,844							0,654	0,190
Gp4	0,620				1,183						0,901	0,282
Gp5	0,395				1,099						0,747	0,352
Gp6	0,394				0,450						0,422	0,028
Gp7		0,868			0,708						0,788	0,080
Gp8		0,650			0,459						0,555	0,095
Gp9		0,592			0,602						0,597	0,005
Gp10		0,223					0,003				0,113	0,110
Gp11		0,357				0,627					0,492	0,135
Gp15		0,211				0,565					0,388	0,177
Gp18			1,493			0,495					0,994	0,499
Gp19			1,743			0,580					1,162	0,581
Gp20				0,100		0,655					0,378	0,278
Gp22			0,032				0,006				0,019	0,013
Gp23			0,493				0,218				0,356	0,137
Gp25			0,797				0,179				0,488	0,309
Gp29				0,246				1,253			0,749	0,504
Gp30									0,824	1,237	1,031	0,206
Gp32									0,939	2,239	1,589	0,650
Gp35				0,130			0,134				0,132	0,002
Gp38				0,247			0,395				0,321	0,074
